# Removal of Mercury from Aqueous Environments Using Polyurea-Crosslinked Calcium Alginate Aerogels

**DOI:** 10.3390/gels11060437

**Published:** 2025-06-06

**Authors:** Evangelia Sigala, Artemisia Zoi, Grigorios Raptopoulos, Elias Sakellis, Aikaterini Sakellari, Sotirios Karavoltsos, Patrina Paraskevopoulou

**Affiliations:** 1 Inorganic Chemistry Laboratory, Department of Chemistry, National and Kapodistrian University of Athens, Panepistimiopolis Zografou, 15771 Athens, Greece; euaggelia.sigala@gmail.com (E.S.); zoiartemisia@gmail.com (A.Z.); grigorisrap@gmail.com (G.R.); 2 Section of Condensed Matter Physics, Department of Physics, National and Kapodistrian University of Athens, 15784 Athens, Greece; e_sakel@phys.uoa.gr; 3 Institute of Nanoscience and Nanotechnology, National Center for Scientific Research “Demokritos”, Agia Paraskevi, 15341 Athens, Greece; 4 Laboratory of Environmental Chemistry, Department of Chemistry, National and Kapodistrian University of Athens, Panepistimiopolis Zografou, 15784 Athens, Greece; esakel@chem.uoa.gr

**Keywords:** biopolymer aerogels, mercury, polymer-crosslinked aerogels, polyurea-crosslinked alginate aerogels, water decontamination

## Abstract

The removal of mercury(II) from aquatic environments using polyurea-crosslinked calcium alginate (X-alginate) aerogels was investigated through batch-type experiments, focusing on low mercury concentrations (50–180 μg·L^−1^), similar to those found in actual contaminated environments. Within this concentration range, the metal retention was very high, ranging from 85% to quantitative (adsorbent dosage: 0.6 g L^−1^). The adsorption process followed the *Langmuir* isotherm model with a sorption capacity of 4.4 mmol kg^−1^ (883 mg kg^−1^) at pH 3.3. Post-adsorption analysis with EDS confirmed the presence of mercury in the adsorbent and the replacement of calcium in the aerogel matrix. Additionally, coordination/interaction with other functional groups on the adsorbent surface may occur. The adsorption kinetics were best described by the pseudo-first-order model, indicating a diffusion-controlled mechanism and relatively weak interactions. The adsorbent was regenerated via washing with a Na_2_EDTA solution and reused at least three times without substantial loss of sorption capacity. Furthermore, X-alginate aerogels were tested for mercury removal from an industrial wastewater sample (pH 7.75) containing 61 μg·L^−1^ mercury (and competing ions), achieving 71% metal retention. These findings, along with the stability of X-alginate aerogels in natural waters and wastewaters, highlight their potential for sustainable mercury removal applications.

## 1. Introduction

Metal pollution of water resources has become a major global concern over the last decades. Classified among the most toxic metals, mercury is detected in all environmental compartments, namely lithosphere, hydrosphere, atmosphere, and biosphere [1]. The primary sources of mercury contamination in aquatic systems include atmospheric deposition, corrosion, urban wastes, agricultural activities, volcanic activity, mining operations (e.g., gold or coal mining), and industrial waste from processes such as chlor-alkali production [1,2,3]. The World Health Organization has established a limit of 0.001 mg L^−1^ for mercury in drinking water [4], which aligns with the corresponding parametric value set by the European Union [5]. Although mercury in surface and ground waters is usually detected at levels not exceeding 0.5 µg L^−1^ [6], several cases have been reported in the literature with concentrations exceeding even 5.5 μg L^−1^, such as in areas with intense volcanic activity (e.g., in Japan) [7].

These elevated mercury concentrations are particularly concerning given its persistence in the environment and its ability to re-enter ecosystems through various exposure pathways, leading to the serious problem of environmental exposure. Mercury can remain in the environment for extended periods, continually re-entering the soil and water and perpetuating exposure risks. This persistence raises concerns over long-term public health implications. Mercury is known to be neurotoxic at elevated doses, also posing significant risks to the cardiovascular, immune, and endocrine systems [8,9,10], with some studies also linking it to some of types of cancer [11].

Within aquatic environments, mercury exists in three oxidation states: elemental mercury Hg(0), Hg(I), and Hg(II), with the highly reactive Hg(II) characterized as the most toxic species [12,13]. Inorganic mercury is primarily ingested from water and food consumption and subsequently accumulated in the kidneys, while elemental mercury crosses the blood–brain barrier [13]. In surface waters, under aerobic conditions and in the presence of bacterial microorganisms, methylmercury ([CH_3_Hg]^+^) can be formed [13], which is a highly toxic, water-soluble mercury species that bioaccumulates in animal tissues and enters the trophic chain.

The significant health risks associated with mercury exposure underscore the urgent need for coordinated public health responses. Regulatory agencies, healthcare providers, and industrial stakeholders, especially in the mining sector, must collaborate to raise awareness, enforce safety standards, and implement robust environmental monitoring systems aimed at reducing mercury pollution and exposure [14]. In tandem with these efforts, the deployment of efficient water treatment technologies plays a critical role in addressing mercury contamination at the source. Numerous techniques have been tested so far for water decontamination from metals, including ion exchange, filtration, chemical precipitation, coagulation, flocculation, reverse osmosis, biosorption, physical adsorption, or a combination thereof [15,16,17]. Among these methods, adsorption stands out for its effectiveness, operational simplicity, and the broad array of available adsorbent materials, which vary in chemical composition, pore structure, and cost. Indeed, adsorbents encompass a variety of materials, including carbon-based materials, clay minerals, zeolites, metal-organic frameworks (MOFs), and numerous nanostructured/nanoporous materials classified as aerogels.

Aerogels can be defined as open non-fluid colloidal or polymer networks expanded with a gas; they can be formed from wet gels by removing all swelling agents without volume reduction or network compaction [18,19,20,21]. These materials have low densities, large surface areas, and high open porosities, including micropores, mesopores and macropores. Due to these properties, aerogels provide a unique platform for adsorption processes, critical for scavenging various pollutants, including metals [22,23,24,25]. Relevant to the subject of the present study, recent studies have demonstrated the potential of various aerogel types (i.e., inorganic, organic, and hybrids thereof) in mercury adsorption, for example, polyacrylamide aerogels (sorption capacity: 18 mg g^−1^) [26], poly (vinyl alcohol)/phytic acid aerogels (40 mg g^−1^) [27], carbon aerogels (7–35 mg g^−1^) [28,29], graphene/diatom silica aerogels decorated with *α*-FeOOH nanoparticles (500 mg g^−1^) [30], sili-ca aerogels (12 mg g^−1^) [26], silica/polyacrylamide aerogels (12–13 mg g^−1^) [26], or sili-ca/gelatin aerogels (209 mg g^−1^) [31]. The latter were also tested for mercury removal from natural water samples (from a pond), demonstrating no loss in removal efficiency. They were successfully regenerated and reused for five cycles without any reduction in capacity. They also showed selectivity for mercury uptake in the presence of co-existing metal ions, such as Pb(II), Cd(II), Cu(II), Zn(II); this can be attributed to the high number of soft Lewis bases (i.e., amine and sulfur-containing functional groups) in the protein side chains, representing ideal ligands for soft metal ions, such as Hg(II).

Relevant to the above, aerogels modified with sulfur-containing groups show increased sorption capacity compared to their unmodified analogues, i.e., silica aerogels modified with mercapto-groups (182 mg g^−1^) [32], thiol-modified nanocellulose aerogels (718.5 mg g^−1^) [33], graphene oxide/MoS_2_ aerogels loaded with Au and Fe_3_O_4_ nanoparticles (1527 mg g^−1^) [34], and MoS_2_ nanoflowers-loaded poly (vinyl alcohol) aerogels (2165 mg g^−1^) [35]. A unique case is the sponge-like CuS aerogels, which exhibited an extremely high sorption capacity of 3667 mg g^−1^ in laboratory samples and 1425 mg g^−1^ in tap and river water [36].

The adsorbents mentioned above have been tested at concentrations in the ppm (mg·L^−1^) range, which are unrealistically high for real-world contaminated water. Among them, silica/gelatin aerogels [31] and CuS aerogels [36] have also been evaluated at trace concentrations (ppb (μg·L^−1^) level), demonstrating nearly quantitative mercury removal. Additionally, graphene oxide/polyethyleneimine aerogels [37] were studied for their performance at mercury concentrations in the ppb range. These aerogels effectively removed mercury from various water sources, including tap water, river water, and seawater, with metal retention ranging from 80 to 91%.

**Scheme 1:**
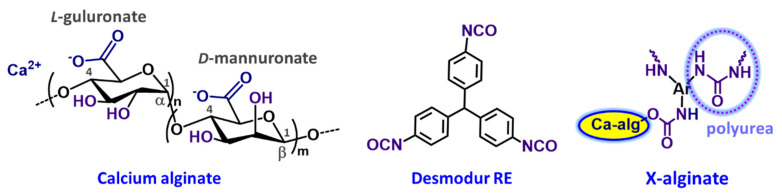
Chemical structures of calcium alginate (a block copolymer of (1→4)-linked *D*-mannuronate and (1→4)-linked *L*-guluronate), triphenylmethane-4,4′,4″-triisocyanate (TIPM; Desmodur RE), and the resulting X-alginate aerogels.

Biopolymer-based aerogels are by design well suited for environmental applications, due to their biocompatibility and the presence of various potential coordination sites (e.g., −COO−, −OH, −NH, −NH_2_). Among these materials, polyurea-crosslinked alginate (X-alginate) aerogels have recently emerged as promising adsorbents for metals in natural waters, including seawater, and wastewaters [38,39,40,41]. These aerogels were prepared following the principles of X-aerogel technology, introduced for silica and expanded to other inorganic oxide [42,43,44] and a few synthetic polymer [45,46,47] aerogels. The synthesis involves reaction of a multifunctional isocyanate with the reactive functional groups on the surface of a pre-formed alginate network, followed by a reaction with water adsorbed onto the alginate network [48,49,50,51]. This process results in the formation of polyurea, which crosslinks the skeletal nanoparticles of the biopolymer skeleton (Scheme 1), thus enhancing the mechanical strength of the material. While no significant morphological changes were observed in the native alginate and the X-alginate network via Scanning Electron Microscopy (SEM), Small-Angle Neutron Scattering (SANS) showed that the type of poly-urea (whether aliphatic or aromatic) fundamentally affects the nanostructure [52], leading to materials with distinct properties and potential for various applications.

Specifically, X-alginate aerogels, prepared via the reaction of triphenylmethane-4,4′,4″-triisocyanate (trade name: Desmodur RE; aromatic triisocyanate) with pre-formed Ca-alginate gels (Scheme 1), exhibit remarkable stability in aquatic environments, including seawater [38]. This stability enables the aerogels to effectively adsorb various metals (Pb(II) [38], U(VI) [39,41], Eu(III) [40], Th(IV) [40], Am(III) [41]) from standard solutions, groundwater, seawater, and wastewater. X-alginate aerogels proved to be highly efficient, even at extremely low pollutant concentrations in the sub-picomolar range [41]. In all cases examined so far, metals were successfully retained within the aerogel matrix, with retention rates ranging from 85% to 99%, and no leakage was observed. Equally noteworthy, the metals could be recovered by treatment with Na_2_EDTA solutions, allowing the adsorbent material to be reused for multiple cycles.

In this study, X-alginate aerogels have been investigated for their ability to capture and remove Hg(II) from aquatic standard solutions in the concentration range 5–2000 μg·L^−1^. Adsorption kinetics and reusability of the adsorbent material were also evaluated. While most literature studies focus on concentrations in the ppm range, which are unrealistically high for real contaminated waters, this work targets very low concentrations (in the ppb range), like those observed in contaminated environments. Additionally, the performance of X-alginate aerogels in decontaminating a wastewater sample was tested in order to demonstrate the potential of these materials for practical applications.

## 2. Results and Discussion

### 2.1. Preparation and Characterization of X-Alginate Aerogel Beads

**Figure 1:**
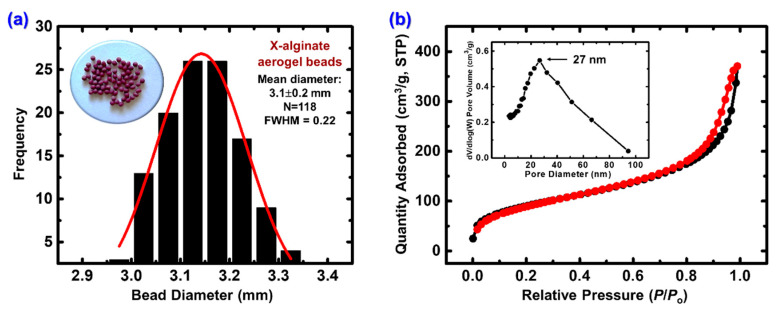
(**a**) Optical image and size distribution of X-alginate aerogel beads. Diameters were measured with ImageJ Version 1.54, and the histogram was generated using OriginPro 9.0. Mean diameter, sample size (N), and full width at half maximum (FWHM) are shown in the Figure. (**b**) N_2_-sorption isotherm of X-alginate aerogel beads. The black line shows adsorption and the red line shows desorption. The inset shows the pore size distribution calculated by the BJH method.

The preparation of X-alginate aerogel beads (Scheme 1) with aromatic polyurea (from Desmodur RE) has been reported in previous studies [38,39,50]. Briefly, preformed calcium alginate gels in the form of beads reacted with Desmodur RE to form polyurea-crosslinked alginate (X-alginate) gels. The polymerization of the triisocyanate to polyurea takes place inside the pores of the preformed biopolymer gels, with the water required for this reaction being adsorbed on the skeleton. This results in interparticle crosslinking by poly-urea, which reinforces the biopolymer network. After the reaction, the beads were dried with SCF CO_2_, yielding X-alginate aerogel beads with a mean diameter of 3.1 ± 0.2 mm (Figure 1a).

X-alginate aerogel beads were characterized with infrared spectroscopy (ATR FTIR; Figure S1), nitrogen porosimetry (Figure 1b; Table S1), and He pycnometry (Table S1). All data agreed with the literature [50] and confirmed the formation of aromatic polyurea on the calcium alginate backbone. In brief, ATR-FTIR spectra (Figure S1) showed the characteristic stretching vibrations of the carboxylate groups coordinated to Ca^2+^ at 1600 (asymmetric) and 1420 (symmetric) cm^−1^, the stretching vibrations of the C–O–C group on the sugar ring at 1084 (asymmetric) and 1034 (symmetric) cm^−1^, the scissoring vibration of the N–H group of the polyurea at 1535 cm^−1^, and the stretching vibrations of the aromatic double bonds at 1508 (asymmetric) and 1413 (symmetric) cm^−1^. The polyurea content of the aerogels was calculated from bulk density (0.109 g cm^−3^) and skeletal density (1.65 g cm^−3^) values (Table S1) [48] and it was found equal to 40% *w*/*w*. Additionally, the calcium content was quantified at 2% *w*/*w*, as measured by ICP-MS. X-alginate aerogel beads are highly porous materials, with a porosity (*Π*) of 93% *v/v* (Table S1). They are also mostly macroporous, as indicated from N_2_ sorption and density measurements. The shape of the N_2_ sorption isotherm (Figure 1b) shows no saturation plateau and a narrow hysteresis loop, indicative of macroporous materials. The BJH pore size distribution (Figure 1b inset) is broad, as expected for materials prepared via diffusion-limited aggregation, with a maximum at 27 nm. The total pore volume calculated from the bulk and skeletal density values (*V*_Total_ = 8.6 cm^3^ g^−1^; Table S1) was significantly higher than the pore volume reported from N_2_ porosimetry for pores with sizes in the range of 1.7 to 300 nm (*V*_1.7–300nm_ = 0.5 cm^3^ g^−1^; Table S1). X-alginate aerogels also show a small amount of microporosity (micropore surface area equal to 16 m^2^ g^−1^), which is an intrinsic property of all aerogel materials prepared using this specific aromatic triisocyanate attributed to its rigid core [38,39,50,53,54,55,56,57,58,59,60].

### 2.2. Evaluation of X-Alginate Aerogels in Terms of Mercury Removal

The X-alginate aerogels used in this study were selected based on the findings of our previous studies on their nanostructure and metal uptake performance in water, natural waters, and wastewaters. Regarding their nanostructure, SANS data for X-alginate aerogels bearing an aliphatic or aromatic polyurea revealed distinct differences [52]. The aromatic polyurea forms a rigid network that grows randomly within the secondary particles of the aerogels, leaving the primary particles accessible to metal ions in solution. In contrast, the aliphatic polyurea coats the aerogel network more uniformly, thereby reducing access to the primary particles. This fundamental structural difference results in a significantly lower adsorption capacity for the aliphatic polyurea-modified aerogels compared to their aromatic counterparts. For example, under identical conditions, sorption capacities for Pb(II) were found to be 20.8 mg g^−1^ (aromatic) and 6.8 mg g^−1^ (aliphatic) [52]. Additionally, due to the rigid structure of the aromatic polyurea, the corresponding X-alginate aerogels exhibit remarkable stability in aquatic environments, including seawater [38].

Therefore, X-alginate aerogels bearing aromatic polyurea have been employed in numerous studies for the removal of metals from aqueous environments, including not only standard solutions but also natural water samples, such as Pb(II) from seawater [38], U(VI) from groundwater and seawater [39,41], Eu(III) [40] and Th(IV) from wastewaters [40], and Am(III) from groundwater and seawater [41]. The most remarkable case was U(VI), where X-alginate aerogels were able to adsorb up to twice their own mass in uranium [39], while they also demonstrated high efficiency at U(VI) and Am(III) concentrations in the sub-picomolar range [41]. In all cases, the X-alginate matrix effectively retained metal ions, achieving retention rates between 85% and 99%, with no detectable leakage. Notably, the adsorbed metals could be efficiently desorbed using Na_2_EDTA solutions, allowing the adsorbent to be regenerated and reused over multiple cycles.

In this study, X-alginate aerogels were evaluated in terms of their capacity to adsorb and remove Hg^2+^ ions from aqueous solutions. The range of concentrations examined varied between 5 and 2000 μg·L^−1^ (2.5 × 10^−5^ and 0.01 mM), representative of the concentrations generally encountered in industrial wastewaters not exceeding 200 μg·L^−1^ [3,61]. The effects of pH, adsorbent dosage, and contact time on the adsorption were investigated, in order to identify optimum conditions and assess the potential of these materials for practical applications. The reusability of the adsorbent was also tested as well as its efficiency in mercury removal from industrial wastewaters (Section 2.3 and 2.4).

#### 2.2.1. Optimal Conditions for Mercury Adsorption

The pH of the aqueous solution plays a critical role in the adsorption process. To achieve maximum adsorption of a specific pollutant on a specific adsorbent, two key factors must be considered: the pH of zero-point charge (pH_ZPC_) of the adsorbent and the speciation diagram of the pollutant.

**Figure 2:**
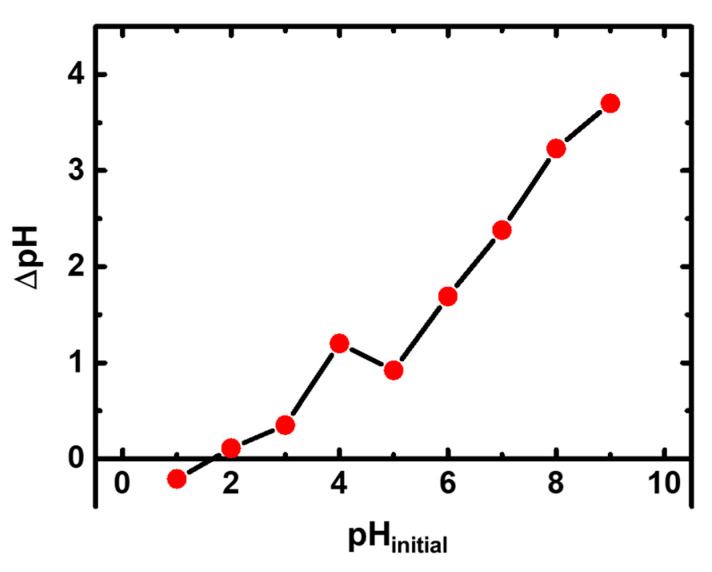
Plot of pH change (ΔpH) versus the initial pH (pH_initial_) for the determination of the pH of zero-point charge (pH_ZPC_) of X-alginate aerogels.

**Figure 3:**
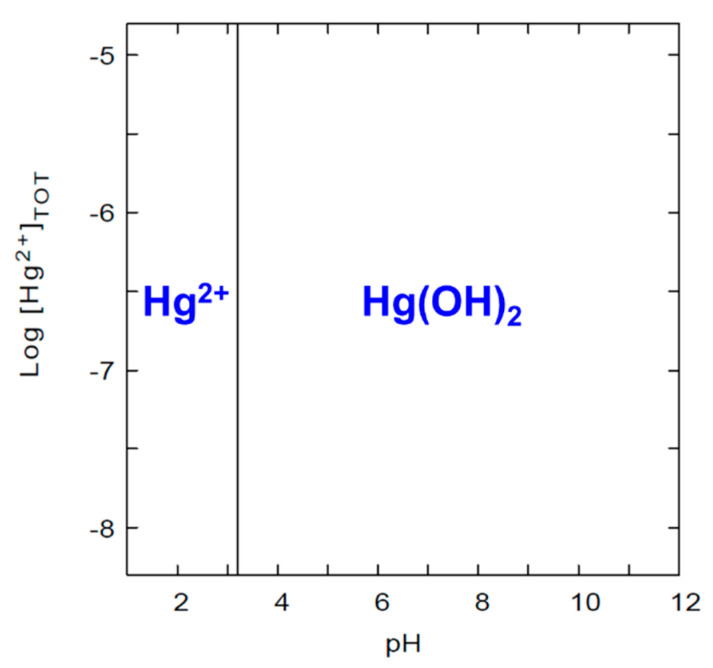
Speciation diagram for Hg(II) as a function of pH under ambient conditions and in the concentration range 5–2000 μg·L^−1^ (2.5 × 10^−5^ and 0.01 mM). Diagram was created using the Medusa-Hydra chemical equilibrium software (https://www.kth.se/che/medusa/, accessed on 26 May 2025) [63].

The pH_ZPC_ of X-alginate aerogels was determined graphically (Figure 2), by the so-called pH-drift method [62] and it was found equal to 1.66. The pH_ZPC_ defines the pH value where the electrical charge of the surface of the material is zero. At pH values lower than the pH_ZPC_, the material carries a positive charge, which could reduce the adsorption of Hg^2+^ due to electrostatic repulsion. In addition, at very low pH values (in our case lower than 1.66), the concentrations of H_3_O^+^ are high and potentially competing with Hg^2+^ for adsorption. At pH values higher than the pH_ZPC_, the material carries a negative charge, thereby eliminating the electrostatic repulsion factor. The speciation diagram of mercury in the range of concentrations studied in this work (Figure 3) shows that Hg^2+^ is the only species present up to pH 3.3, whereas no cationic species are present at pH exceeding 3.3.

**Figure 4:**
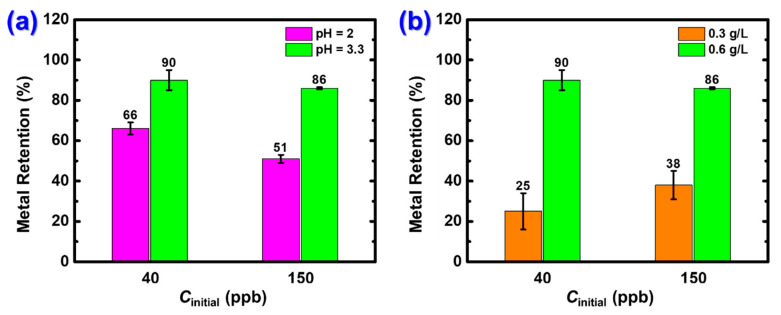
Effect on Hg^2+^ removal from aqueous solutions using X-alginate aerogels of (**a**) pH (25 °C; adsorbent dosage 0.6 g L^−1^; agitation rate 150 rpm; contact time 24 h) and (**b**) adsorbent dosage (25 °C; pH 3.3; agitation rate 150 rpm; contact time 24 h). The initial Hg(II) concentrations were 45 ± 4 and 151 ± 15 μg·L^−1^.

Based on the above, the effect of pH on Hg^2+^ adsorption by X-alginate aerogels was studied at pH values 2.0 and 3.3 at two different mercury concentrations. More specifically, X-alginate aerogel beads were added to Hg^2+^ solutions (0.6 g of aerogel per L of solution) with concentrations equal to 40 and 150 μg·L^−1^ (2 × 10^−5^ and 7.5 × 10^−3^ mM, respectively) at 25 °C for 24 h. The concentration of the solutions was measured using CVAAS. The results, presented in Figure 4a, show that the metal retention was lower at pH 2 compared to that at pH 3.3. Therefore, the rest of the experiments were conducted at pH 3.3. In addition to the above, previous studies have shown that the optimum pH for adsorption of mercury using calcium alginate hydrogel beads is 3–3.6 [64].

Furthermore, two different adsorbent dosages, 0.3 and 0.6 g L^−1^, were tested, following the same procedure as previously described. As shown in Figure 4b, metal retention was significantly higher at the adsorbent dosage of 0.6 g L^−1^, and this dosage was used for the rest of the experiments.

#### 2.2.2. Mercury Adsorption Isotherm

**Figure 5:**
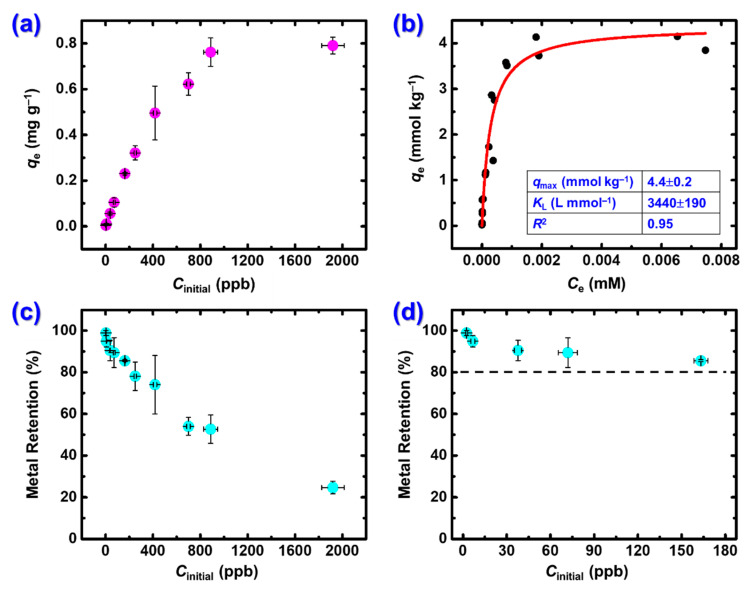
(**a**) Mercury adsorption on X-alginate aerogels (*q*_e_; mg g^−1^) plotted against the initial Hg^2+^ concentration (*C*_initial_; μg·L^−1^). (**b**) Mercury adsorption on X-alginate aerogels (*q*_e_; mmol kg^−1^) plotted against Hg^2+^ concentration at equilibrium (*C*_e_; mM) and *Langmuir* sorption isotherm. (**c**) Hg^2+^ retention (%) on X-alginate aerogels plotted against the initial Hg^2+^ concentration (*C*_initial_; μg·L^−1^). (**d**) Frame (**c**) has been expanded to more clearly show the Hg^2+^ retention (%) at concentrations lower than 180 μg·L^−1^, which are more relevant for environmental samples. Experimental conditions: initial Hg(II) concentrations 5–2000 μg·L^−1^ (2 × 10^−5^ to 0.01 mM); 25 °C; pH 3.3; adsorbent dosage 0.6 g L^−1^; agitation rate 150 rpm; contact time 24 h.

Based on the results reported in Section 2.2.1, the adsorption capacity of X-alginate aerogels for Hg^2+^ was studied with batch-type experiments in the range of concentrations from 5–2000 μg·L^−1^ (2.5 × 10^−5^ to 0.01 mM) at 25 °C, pH 3.3, and an adsorbent dosage of 0.6 g L^−1^. The contact time was set to 24 h to ensure that the system has reached equilibrium. The results are presented graphically in Figure 5. The isothermal data were fitted using the *Langmuir* (Equation (1)), *Freundlich* (Equation (2)) and *Dubinin–Radushkevich (D-R*; Equation (3)) models, where *q*_e_ (mmol kg^−1^) and *C*_e_ (mM) are the mercury uptake and the solution concentration at equilibrium, respectively, *q*_max_ (mmol kg^−1^) is the adsorption capacity of X-alginate beads, *K*_L_ (L mmol^−1^), *K*_F_, and *K*_D_ (mol^2^ kJ^−2^) are the *Langmuir*, *Freundlich*, and *D–R* constants, respectively, *n* is an empirical constant related to the heterogeneity of the adsorbent surface, and *ε* (kJ mol^−1^) is the Polanyi potential (Equation (4)). The corresponding plots and derived parameters are presented in Figure 5b (*Langmuir*), Figure S2a (*Freundlich*), and Figure S2b (*D–R*).

(1) $$q_{e}=\frac{q_{max}K_{L}C_{e}}{1+K_{L}C_{e}}$$

(2) $$q_{e}=K_{F}{C_{e}}^{1/n}$$

(3) $$q_{e}=q_{max}e^{-K_{D}\varepsilon^{2}}$$

(4) $$\varepsilon =RT\ln(1+\frac{1}{C_{e}})$$

The adsorption reaches a plateau for initial concentrations exceeding 840 μg·L^−1^ (Figure 5a) with *q*_e_ values in the range of 790 ± 36 mg Hg^2+^ per g of X-alginate aerogel (3.9 ± 0.2 mmol kg^−1^). The retention (%) of Hg^2+^ on the adsorbent decreases as the initial concentrations increase (Figure 5c). No leakage could be observed from the material after the adsorption, at least for 24 h. At concentrations lower than 180 μg·L^−1^, which are more relevant to environmental samples, the metal retention is very high, ranging from 85% to quantitative (Figure 5c).

The adsorption data were best fitted with the *Langmuir* model (*R*^2^ = 0.95 versus *R*^2^ = 0.85 for the *Freundlich* model and *R*^2^ = 0.90 for the *D–R* model). The calculated *q*_max_ is equal to 4.4 ± 0.2 mmol Hg^2+^ per kg of X-alginate aerogel (883 mg kg^−1^). This value is similar to that found experimentally (3.9 ± 0.2 mmol Hg^2+^ per kg of X-alginate aerogel) and it is notable that this value has been reached for an initial mercury concentration equal to 840 μg·L^−1^ already (Figure 5a). This sorption capacity is higher than that of graphene oxide/polyethyleneimine aerogels (90 mg kg^−1^) [37]. In terms of metal retention, all aerogels studied at ppb-level concentrations—silica/gelatin [31], CuS [36], graphene oxide/polyethyleneimine [37], and X-alginate—achieved mercury removal above 90% at very low concentrations, up to 50 μg·L^−1^.

Previous studies have demonstrated that X-alginate aerogels can be very practical adsorbents, because their adsorption capacity per volume can be very high. That was showcased for U (VI) [39], where X-alginate aerogels by far outperformed all other aerogel materials that had been used for the same purpose. As an example, if X-alginate aerogels (*q*_max_ = 2023 g kg^−1^; *ρ*_b_ = 150 mg cm^−3^) [39] and Al_2_O_3_/MgO aerogels (*q*_max_ = 1046.9 g kg^−1^; *ρ*_b_ = 18.89 mg cm^−3^) [65] were used for the removal of 1 g of U (VI) from water, the mass of X-alginate aerogel needed would be 2 times less than the mass of Al_2_O_3_/MgO aerogel, but the corresponding volume would be 15 times smaller. In the case of mercury, the adsorption capacity per volume of X-alginate aerogels is equal to 96 mg cm^−3^, which means that for the decontamination of 1 L of water containing a mercury load close to the maximum typically encountered in natural samples (i.e., 180 μg·L^−1^), less than 2 cm^3^ (1.9 cm^3^) of X-alginate aerogel are required.

#### 2.2.3. Characterization of X-Alginate Beads After Mercury Adsorption

The results obtained from the batch adsorption tests are in agreement with those of our previous studies on the adsorption of Pb(II) [38], U(VI) [39], Eu(III), and Th(IV) [40], indicating inner-sphere complexation and saturation of the coordination sites available for interaction with the metal ions. Coordination of Hg^2+^ to the carboxylate groups of the alginate is realized by replacing Ca^2+^, as evidenced by the EDS data (Figure S3, Table 1). The Hg/Ca ratio (both atomic and by weight) increases with rising Hg^2^⁺ concentration, supporting this claim. It is important to note that the EDS experiments were conducted with initial mercury concentrations in the ppm range, as at lower concentrations, adsorbed mercury could not be accurately quantified or even detected. If there were no replacement of Ca^2^⁺, the Hg/Ca ratio after adsorption would be lower than the experimentally observed value, even if mercury adsorption was complete. For instance, in the experiment with an initial mercury concentration of 50 mg·L^−1^, the Hg/Ca ratio would be 0.84 (calculated as 0.012 mol Hg^2^⁺ in solution/0.015 mol Ca^2^⁺ in the aerogel = 0.84), which is substantially lower than the experimental value of 3.50 (Table 2). Additionally, Hg^2^⁺ may coordinate with other functional groups, such as –NH and/or –OH, on the surface of the aerogel. Previous studies with biopolymer-based adsorbents have shown that amine groups enhance the sorption capacity for mercury [66,67]; for example, glutaraldehyde-crosslinked alginate gels containing chitosan had a sorption capacity over 20 times higher than that of the corresponding alginate gels [66].

#### 2.2.4. Time-Resolved Adsorption Experiments

**Figure 6:**
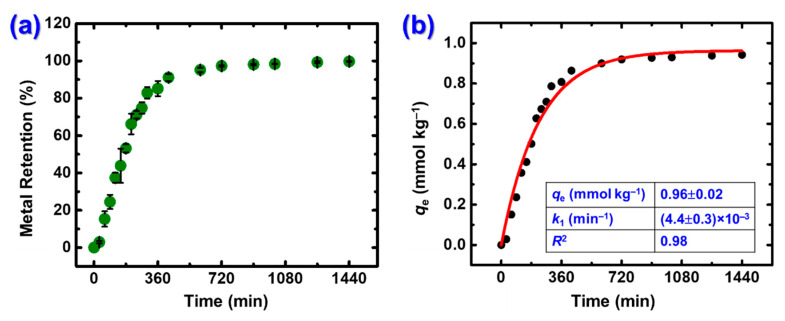
(**a**) Effect of contact time on Hg^2+^ removal from an aqueous solution using X-alginate aero-gels. Experimental conditions: initial Hg(II) concentration 116 ± 20 μg·L^−1^; 25 °C; pH 3.3; adsorbent dosage 0.6 g L^−1^; agitation rate 150 rpm; contact time 24 h. (**b**) Pseudo-first order kinetic model fit.

The effect of contact time was studied following the same procedure as described above, obtaining samples from the solution at regular time intervals. The initial Hg^2+^ concentration was 116 ± 20 μg·L^−1^, within the range reported for natural environmental polluted samples. As shown in Figure 6a, mercury adsorption increased rapidly within the first 6 h, reaching 85% metal retention, and became nearly quantitative within the next 4 h. The adsorption rate decreased after the first 6 h, due to the gradual occupation of sites available for Hg^2+^ coordination and to the decreasing concentration of Hg^2+^ in solution. To investigate the adsorption kinetics, the experimental data were fitted to three theoretical models: pseudo first-order, pseudo second-order, and intraparticle diffusion kinetic models (Equations (5)–(7)), where *q*_t_ and *q*_e_ (mmol kg^−1^) represent mercury uptake at time *t* and at equilibrium, respectively, while *k*_1_ (min^−1^), *k*_2_ (kg mmol^−1^ min^−1^) and *k*_i_ (mmol kg^−1^ min^−1/2^) are the corresponding rate constants. Fitting of experimental data is shown in Figure 6b (pseudo-first order) and Figure S4 (a: pseudo-second order and b: intraparticle diffusion) and the derived parameters are presented in Table 2. Better adaptability of the curve and higher *R*^2^ value are observed for the pseudo-first order model, suggesting a diffusion-controlled process and relatively weak interactions (van der Waals forces or electrostatic interactions). Moreover, the *q*_e_ value calculated from that model (0.96 mmol kg^−1^) is closer to the experimental value (0.82 mmol kg^−1^; Figure 5b), further confirming that this model fits better to the experimental data.

(5) $$q_{t}=q_{e}{(1-e}^{{-k}_{1}t})$$

(6) $$q_{t}=\frac{{q_{e}}^{2}k_{2}t}{1+q_{e}k_{2}t}$$

(7) $$q_{t}={k_{i}t^{1/2}+q}_{e}$$

### 2.3. Reusability of the Adsorbent

**Figure 7:**
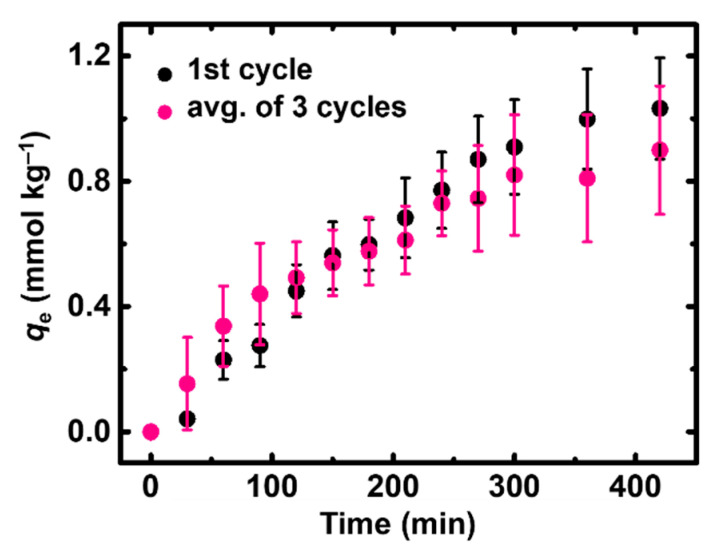
Adsorption of Hg^2+^ (mmol kg^−1^) using X-alginate aerogels from an aqueous solution with initial concentration 120 ± 12 μg·L^−1^. Experimental conditions: 25 °C; pH 3.3; adsorbent dosage 0.6 g L^−1^; agitation rate 150 rpm. Black dots: data from Figure 6b; average of three measurements with fresh X-alginate aerogels. Pink dots: average of three measurements with reused X-alginate aerogels.

**Figure 8:**
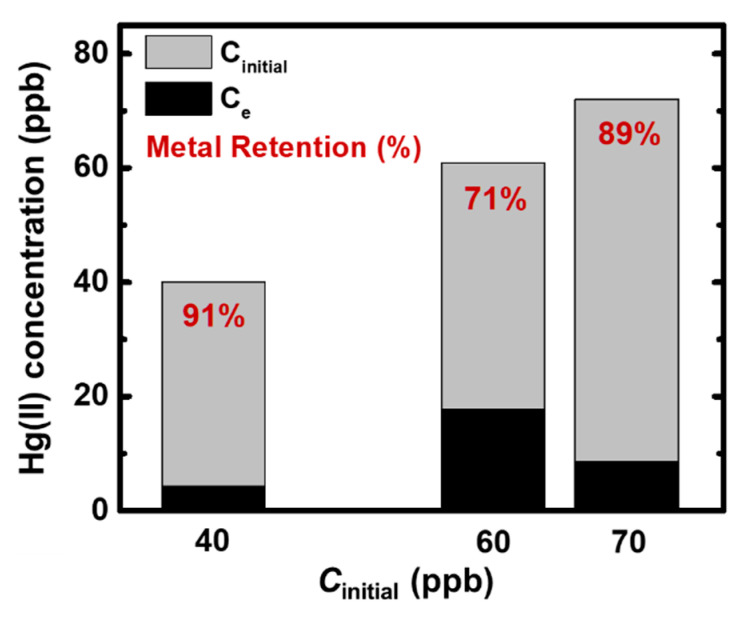
Adsorption of Hg(II) from industrial wastewater using X-alginate aerogels (initial Hg(II) concentration: 61 ± 1 μg·L^−1^; 25 °C; pH 7.74; adsorbent dosage 0.6 g L^−1^; agitation rate 150 rpm; contact time 24 h) and comparison with adsorption data from two standard solutions (initial Hg(II) concentrations: 38 ± 2 and 72 ± 7 μg·L^−1^; 25 °C; pH 3.3; adsorbent dosage 0.6 g L^−1^; agitation rate 150 rpm; contact time 24 h—data from Figure 5), as indicated.

The reusability of X-alginate aerogels was studied using a 120 μg·L^−1^ (0.0006 mM) Hg^2+^ solution (Figure 7). This concentration is representative of industrial wastewaters characterized by mercury levels not exceeding 200 μg·L^−1^ [3,61]. In addition, in the industrial wastewater sample used in this study, the initial Hg(II) concentration was 61 μg L^−1^ (Section 2.4 and Figure 8). Therefore, the concentration of 120 μg L^−1^ was selected as a representative mid-range value within the range mentioned above. After completion of the adsorption (1st cycle; pH 3.3, 24 h), the X-alginate beads were removed from the solution, and washed thoroughly with a Na_2_EDTA solution and subsequently with water. The recovery of Hg(II) was quantitative. Then, the beads were added in a fresh Hg^2+^ solution of the same concentration (120 ± 12 μg·L^−1^) and under identical experimental conditions, to complete the 2nd cycle. The same procedure was repeated for a total of three cycles. In each cycle, the solution was sampled at regular time intervals and the concentration of Hg^2+^ remaining in the solution was monitored for 400 min. As demonstrated in Section 2.2.4, adsorption is nearly quantitative by that time and remains unchanged thereafter, indicating that equilibrium has already been reached. The results (Figure 7) showed that X-alginate beads could be reprocessed and reused at least three times without significant loss of their adsorption capacity.

### 2.4. Mercury Removal from Industrial Wastewaters

Mercury uptake experiments were also conducted with an industrial wastewater sample, in order to investigate the applicability of X-alginate aerogel beads in real contaminated samples. From our previous studies, X-alginate aerogels have shown extremely high stability (i.e., no shrinkage, swelling, or disintegration) in natural waters and wastewaters [38,39,40,41]. The pH of the industrial wastewater was 7.74 and in addition to Hg(II) (61 ± 1 μg·L^−1^) it contained several other metal ions at various concentrations (μg·L^−1^), among which were nickel (310), iron (16.8), chromium (13.8), and cobalt (9.82) (Table S2). The uptake of Hg(II) was equal to 0.068 mg g^−1^ (0.34 mmol kg^−1^) and the metal retention was equal to 71 ± 7% (Figure 8). By comparison to the results from Hg(II) standard solutions at concentrations close to that of Hg(II) in the wastewater sample (i.e., 38 ± 2 and 72 ± 7 μg·L^−1^), the metal retention was lower, as expected, due to different experimental conditions. Specifically, in alkaline solutions (wastewater sample), the dominant species is Hg(OH)_2_, and not Hg^2+^ as in acidic ones (standard solutions; Figure 3). In addition, the competing ions contained in the wastewater sample may also be adsorbed on X-alginate beads, reducing the number of available coordination sites. The results of the present work point to a selectivity of X-alginate aerogels towards Hg(II), which requires further investigation.

## 3. Conclusions

Polyurea-crosslinked calcium alginate (X-alginate) aerogels were investigated for their ability to remove Hg(II) from aquatic environments. These aerogels contain 2% *w*/*w* calcium and 40% *w*/*w* polyurea. They have low bulk density (0.109 g cm^−3^), high specific surface area (322 m^2^ g^−1^), and high porosity (93% *v*/*v*), including macropores (mostly), mesopores, and micropores. Mercury uptake from water was investigated through batch-type experiments, with concentrations in the range of 5–2000 μg·L^−1^, including levels (50–180 μg·L^−1^) typical of real-world contaminated settings. The optimal conditions for mercury removal were found at pH 3.3 and an adsorbent dosage of 0.6 g L^−1^. Within this concentration range, metal retention was notably high, ranging from 85% to quantitative. The adsorption process followed the *Langmuir* isotherm model, with a sorption capacity of 4.4 mmol kg^−1^ (883 mg kg^−1^).

Post-adsorption analysis using energy-dispersive X-ray spectroscopy (EDS) confirmed the presence of mercury in the adsorbent and also confirmed that Hg(II) replaces Ca(II) in the aerogel matrix, while coordination/interaction of mercury with amine and hydroxy groups on the surface of X-alginate aerogels can also take place. The adsorption kinetics was best described by the pseudo-first-order model, suggesting a diffusion-controlled mechanism and relatively weak interactions.

The adsorbent demonstrated good reusability, with no significant loss in sorption capacity after at least three cycles of regeneration using a Na_2_EDTA solution. X-alginate aerogels were also tested for mercury removal from an industrial wastewater sample (pH 7.75) containing 61 μg·L^−1^ of mercury, achieving a high metal retention of 71% in the presence of competing ions. Overall, the stability, reusability, and high mercury removal efficiency of X-alginate aerogels make them a promising and sustainable solution for both natural water and wastewater treatment and decontamination.

Further studies will focus on a comprehensive evaluation of the adsorption performance of X-alginate aerogels for mercury, considering additional variables such as temperature effects, coexisting ion concentrations, and gradients in initial Hg(II) concentrations. Their effectiveness will also be assessed in real wastewater samples and contaminated natural waters. In parallel, surface modification strategies, such as the attachment of complexing moieties (e.g., amine and hydroxy groups), will be explored to enhance sorption capacity and selectivity towards mercury.

## 4. Experimental Section

### 4.1. Materials and Methods

Sodium alginate (PROTANAL LF 240 D; G 30–35%) was purchased from FMC. CaCl_2_·2H_2_O, acetone, and acetonitrile (MeCN; HPLC grade) were purchased from Fisher Scientific. The mercury standard solution (1000 mg·L^−1^) was purchased from Merck. Desmodur RE (27% w/w triphenylmethane-4,4′,4″-triisocyanate (TIPM) solution in ethyl acetate) was generously provided by Covestro AG. The mercury standard solution was purchased from Merck. Disodium ethylenediaminetetraacetate (Na_2_EDTA) was purchased from Lach-Ner. All solvents and reagents were used as received.

Supercritical fluid (SCF) drying was performed in an autoclave (Model E3100, Quorum Technologies, East Sussex, UK). The gels were placed in the autoclave at 12 °C and covered with acetone. Liquid CO_2_ was then allowed in the autoclave; acetone was drained out as it was displaced by liquid CO_2_ (5×; 1 per 30 min). The temperature of the autoclave was increased to 45 °C and maintained for 1 h. Finally, the pressure was slowly released, allowing supercritical CO_2_ to escape as a gas.

N_2_-sorption measurements were performed on a Micromeritics Tristar II 3020 surface area and porosity analyzer (Micromeritics, Norcross, GA, USA). Samples were degassed at 80 °C for 24 h using Micromeritics VacPrep 061. Skeletal densities (*ρ*_s_) were determined by He pycnometry using a Micromeritics AccuPyc II 1340 pycnometer. Bulk densities (*ρ*_b_) of the samples were calculated from their weight and natural dimensions.

ATR-FTIR spectra were obtained with a Shimadzu FTIR IR Affinity-1 spectrometer equipped with an ATR Shimadzu QATR10 single-reflection attachment. Spectra were recorded in the wavelength range 400–4000 cm^−1^. EDS spectra were measured with an FEI Quanta Inspect microscope operating at 10 kV accelerating voltage.

pH measurements were performed using a commercial glass electrode, calibrated prior to and after each experiment using a series of buffer solutions. Concentrations of Hg(II) in aqueous solutions were measured by Cold Vapor Atomic Absorption Spectrometry, Varian VGA-77 (Varian, Mulgrave, Australia), following appropriate dilution of the samples.

### 4.2. Preparation of X-Alginate Aerogel Beads

X-alginate aerogel beads were prepared and characterized as described before [50]. Briefly, an aqueous solution of sodium alginate (2% *w*/*w*) was added dropwise, using a 25 mL burette, to a 0.2 M solution of CaCl_2_ under mild magnetic stirring. Spherical hydrogel alginate beads were formed instantly, and they were left to age for 24 h. Then, the beads were stepwise solvent-exchanged with MeCN/H_2_O mixtures (30, 60, 90% *v*/*v*) and finally with MeCN (4×). Subsequently, the beads were kept in a solution of TIPM in ethyl acetate/MeCN (0.75 M) for 24 h at room temperature and for 72 h at 70 °C. Afterward, the beads were solvent-exchanged with acetone (3×) and dried from supercritical CO_2_ to the corresponding aerogels (X-alginate aerogels).

### 4.3. Determination of the pH of Zero-Point Charge (pH_ZPC_) of X-Alginate Aerogels

The pH of zero-point charge (pH_ZPC_) of X-alginate aerogels was determined graphically using the pH-drift method [62]. A series of NaCl solutions (0.1 M) with initial pH values in the range of 1–9 (pH_initial_) were prepared by adjusting the pH using NaOH and HCl solutions (0.1M). X-alginate aerogel beads (~3.5 mg) were added to each one of the NaCl solutions (30 mL), which were kept under stirring at room temperature for 24 h. Afterwards, the final pH (pH_final_) of the solutions was measured and ΔpH (ΔpH = pH_final_ − pH_initial_) was plotted against the initial pH. The pH_ZPC_ is the pH at which ΔpH = 0.

### 4.4. Mercury Uptake from Aqueous Solutions

Glassware and plastic vials used throughout were previously cleaned with supra-pure HNO_3_ 10% (Merck) and rinsed with ultrapure water of 18.2 MΩ/cm (Millipore, Bedford, MA, USA). X-alginate aerogel beads (0.03 g) accurately weighed were added to aqueous solutions (50 mL; adsorbent dosage: 0.6 g L^−1^) of varying Hg^2+^ concentrations in the range 5 to 2000 μg·L^−1^ (2.5 × 10^−5^ to 0.01 mM). Preparation of Hg^2+^ standard solutions was carried out after appropriate dilutions of the corresponding stock standard solution (1000 mg·L^−1^). All required solutions were prepared using class A volumetric glassware. The pH was adjusted at 2.0 or 3.3 with a NaOH solution (5% *v*/*v*). The contact time, under magnetic stirring, was set at 24 h and the temperature was maintained at 20 °C. The determination of Hg(II) in the solutions (prior to the adsorption and following its completion) was carried out by Cold Vapor Atomic Absorption Spectrometry (CVAAS), employing a Varian SpectrAA 200 with VGA-77 instrument (Varian, Mulgrave, Australia) (LOD equal to 0.15 μg·L^−1^), following appropriate dilution of the samples.

The amount of Hg(II) adsorbed per aerogel mass unit at time *t*, *q*_t_ (mmol kg^−1^), was calculated using Equation (8), where *C*_i_ (mM) and *C*_t_ (mM) are the initial and the final (at time *t*) Hg^2+^ concentrations, *V* (L) is the volume of the solution, and *m* (g) is the weight of X-alginate aerogels. Experiments were performed in triplicate and mean values were used for data evaluation.

(8) $$q_{t}=\frac{(C_{i}-C_{t})V}{m}$$

### 4.5. Reusability of X-Alginate Adsorbent

The recovery of Hg(II) from X-alginate beads was performed via extraction with aqueous Na_2_EDTA solutions (0.1 M, pH 10). After the adsorption tests, X-alginate beads were kept in a Na_2_EDTA solution for 24 h, then thoroughly washed with water (6× in total; twice a day) before being reused.

### 4.6. Mercury Uptake from Wastewaters

A procedure similar to that described in Section 2.4 was used for the study of Hg(II) uptake from industrial wastewater samples. For this purpose, a representative wastewater sample was examined. The pH was measured equal to 7.74, while the metal content was analyzed through inductively coupled plasma—mass spectrometry (ICP-MS), employing a Thermo Scientific ICAP Qc (Waltham, MA, USA) instrument. The results obtained are reported in Table S2.

## Figures and Tables

**Table 1:** EDS data showing the calcium and mercury content of X-alginate beads after Hg^2+^ adsorption from aqueous solutions in the concentration range 0.01–50 mg·L^−1 a^.

Initial Hg^2+^ Concentration (mg·L^−1^)	Hg^2+^ in Solution (mmol)	CaK (% *w*/*w*)	CaK (% Atomic)	HgL (% *w*/*w*)	HgL (% Atomic)	Atomic Ratio Hg/Ca
0.01				cannot be quantified		
5	0.001	80.58	95.40	19.42	4.60	0.05
10	0.002	60.68	88.54	39.32	11.46	0.13
20	0.005	50.12	83.41	49.88	16.59	0.20
50	0.012	5.39	22.20	94.61	77.80	3.50

^a^ Volume of solution: 50 mL; aerogel dosage: 0.6 mg L^−1^.

**Table 2:** Mercury uptake (*q*_e_) by X-alginate aerogel beads and rate constants *k*_1_, *k*_2_, and *k*_i_ derived from the pseudo first-order, the pseudo second-order, and the intraparticle diffusion kinetic models.

**Pseudo first-order**	*q*_e_ (mmol kg^−1^)	0.96 ± 0.02
	*k*_1_ (min^−1^)	(4.4 ± 0.3) × 10**^−^**^3^
	*R* ^2^	0.98
**Pseudo second-order**	*q*_e_ (mmol kg^−1^)	1.17 ± 0.06
	*k*_2_ (kg mmol^−1^ min^−1^)	(4.0 ± 0.8) × 10**^−^**^3^
	*R* ^2^	0.95
**Intraparticle diffusion**	*q*_e_ (mmol kg^−1^)	0.10 ± 0.07
	*k*_2_ (mmol kg^−1^ min^−1/2^)	0.028 ± 0.003
	*R* ^2^	0.8

## Data Availability

The original contributions presented in this study are included in the article/Supplementary Material. Further inquiries can be directed to the corresponding authors.

## Supplementary Materials

The following supporting information can be downloaded at: https://www.mdpi.com/article/10.3390/gels11060437/s1. Figure S1. ATR-FTIR spectra of X-alginate aerogel beads. The characteristic peaks for the Ca-alginate skeleton are noted with blue and the ones for polyurea (PUA) are noted with red. Figure S2. *Freundlich* (a) and *Dubinin–Radushkevich* (*D–R*; b) sorption isotherms of Hg^2+^ on X-alginate aerogels. Experimental conditions: initial Hg^2+^ concentrations 5–2000 μg·L^−1^ (2 × 10^−5^ to 0.01 mM); 25 °C; pH 3.3; adsorbent dosage 0.6 g L^−1^; agitation rate 150 rpm; contact time 24 h. Figure S3. EDS spectra of X-alginate beads after adsorption from solutions with initial Hg^2+^ concentrations equal to 5, 10, 20, and 50 mg·L^−1^, as indicated. Figure S4. Pseudo-second order (a) and intraparticle diffusion (b) kinetic model fit for Hg^2+^ adsorption on X-alginate aerogel beads. Table S1. Selected material properties of X-alginate aerogel beads. Table S2. Metals contained in the industrial wastewater used in this study.
