# Supplementary material for: Removal of Mercury from Aqueous Environments Using Polyurea-Crosslinked Calcium Alginate Aerogels
Source: Gels. 2025 Jun 6;11(6):437. doi: 10.3390/gels11060437 (PMC12192515; doi:10.3390/gels11060437)
Supplement: Supplementary file 1 [file gels-11-00437-s001.zip › gels-3640649-supplementary.pdf]

SUPPORTING INFORMATION

Removal of Mercury from Aqueous Environments using Polyurea-Crosslinked Calcium Alginate Aerogels

Evangelia Sigala[1], Artemisia Zoi[1], Grigorios Raptopoulos[1], Elias Sakellis[2,3], Aikaterini Sakellari[4], Sotirios Karavoltsos[4,*], Patrina Paraskevopoulou[1,*]

[1]Inorganic Chemistry Laboratory, Department of Chemistry, National and Kapodistrian University of Athens, Panepistimiopolis Zografou, 15771 Athens, Greece

[2]Section of Condensed Matter Physics, Department of Physics, National and Kapodistrian University of Athens, Athens, 15784, Greece

[3]Institute of Nanoscience and Nanotechnology, National Center for Scientific Research "Demokritos", Agia Paraskevi, Athens, 15341, Greece

[4]Laboratory of Environmental Chemistry, Department of Chemistry, National and Kapodistrian University of Athens, Panepistimiopolis Zografou, 15784 Athens, Greece

* Correspondence: paraskevopoulou@chem.uoa.gr (P.P.); skarav@chem.uoa.gr (S.K.)

Table of Contents

Figure S1. ATR-FTIR spectra of X-alginate aerogel beads. The characteristic peaks for the Ca-alginate skeleton are noted with blue and the ones for polyurea (PUA) are noted with red. 3

Figure S2. *Freundlich* (a) and *Dubinin–Radushkevich* (*D-R*; b) sorption isotherms of Hg^{2+} on X-alginate aerogels. Experimental conditions: initial Hg^{2+} concentrations 5-2000 μg·L⁻¹ (2×10⁻⁵ to 0.01 mM); 25 °C; pH 3.3; adsorbent dosage 0.6 g L⁻¹; agitation rate 150 rpm; contact time 24 h. 3

Figure S3. EDS spectra of X-alginate beads after adsorption from solutions with initial Hg^{2+} concentrations equal to 5, 10, 20 and 50 mg·L⁻¹, as indicated. 4

Figure S4. Pseudo-second order (a) and intraparticle diffusion (b) kinetic model fit for Hg^{2+} adsorption on X-alginate aerogel beads. 4

Table S1. Selected material properties of X-alginate aerogel beads. 5

Table S2. Metal concentrations in the industrial wastewater used in this study. 5

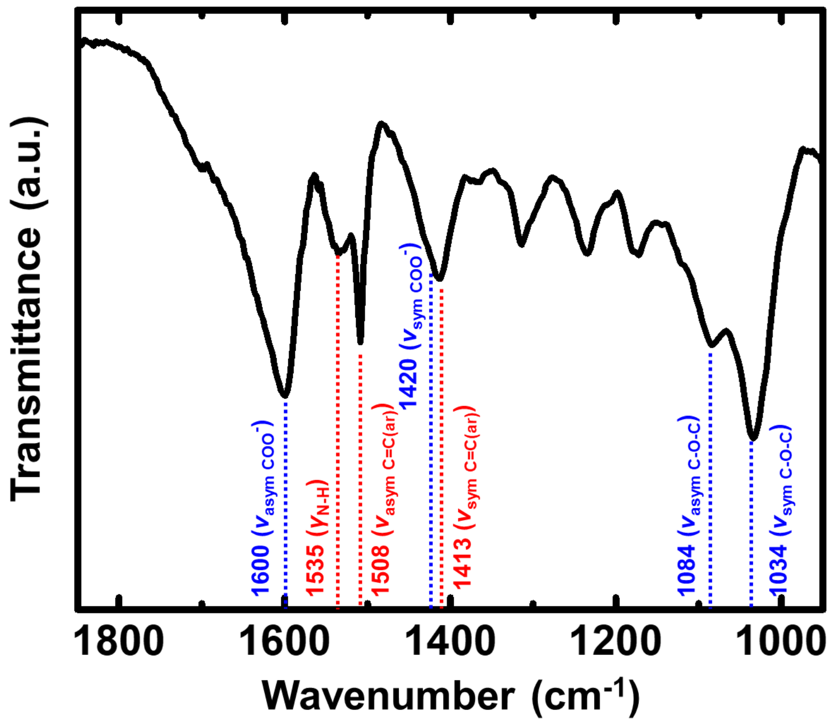

Figure S1. ATR-FTIR spectra of X-alginate aerogel beads. The characteristic peaks for the Ca-alginate skeleton are noted with blue and the ones for polyurea (PUA) are noted with red.

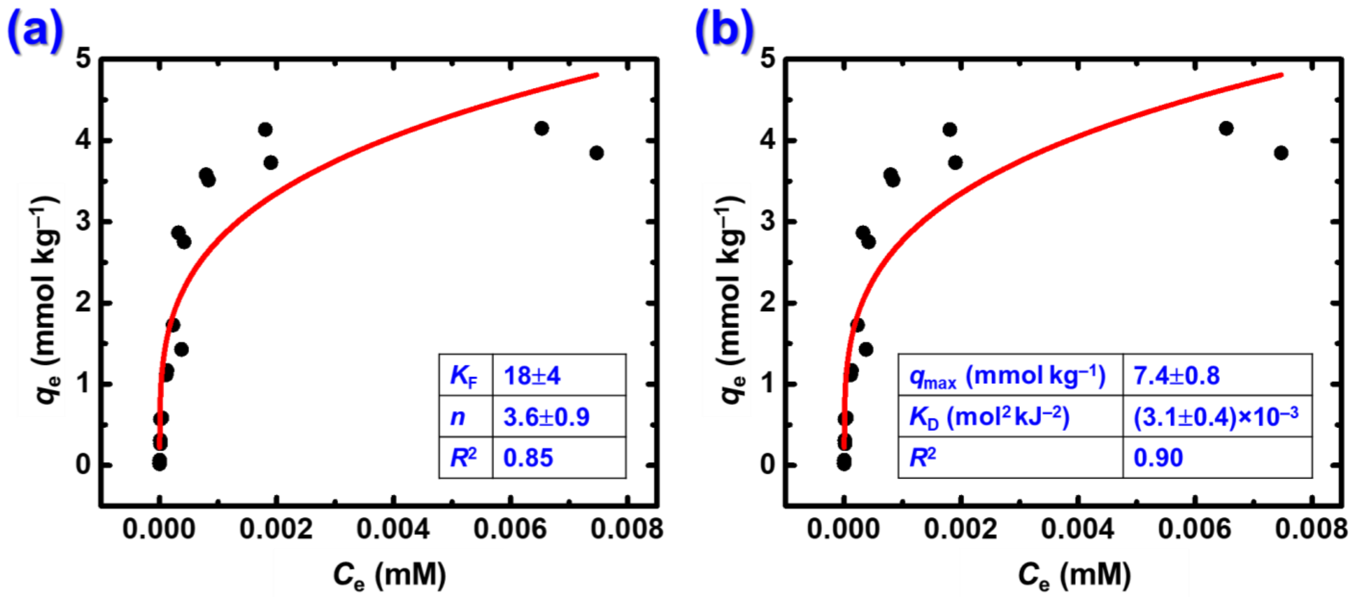

Figure S2. *Freundlich* (a) and *Dubinin–Radushkevich* (*D-R*; b) sorption isotherms of Hg^{2+} on X-alginate aerogels. Experimental conditions: initial Hg^{2+} concentrations 5-2000 μg·L^{-1} (2×10^{-5} to 0.01 mM); 25 ^{o}C; pH 3.3; adsorbent dosage 0.6 g L^{-1}; agitation rate 150 rpm; contact time 24 h.

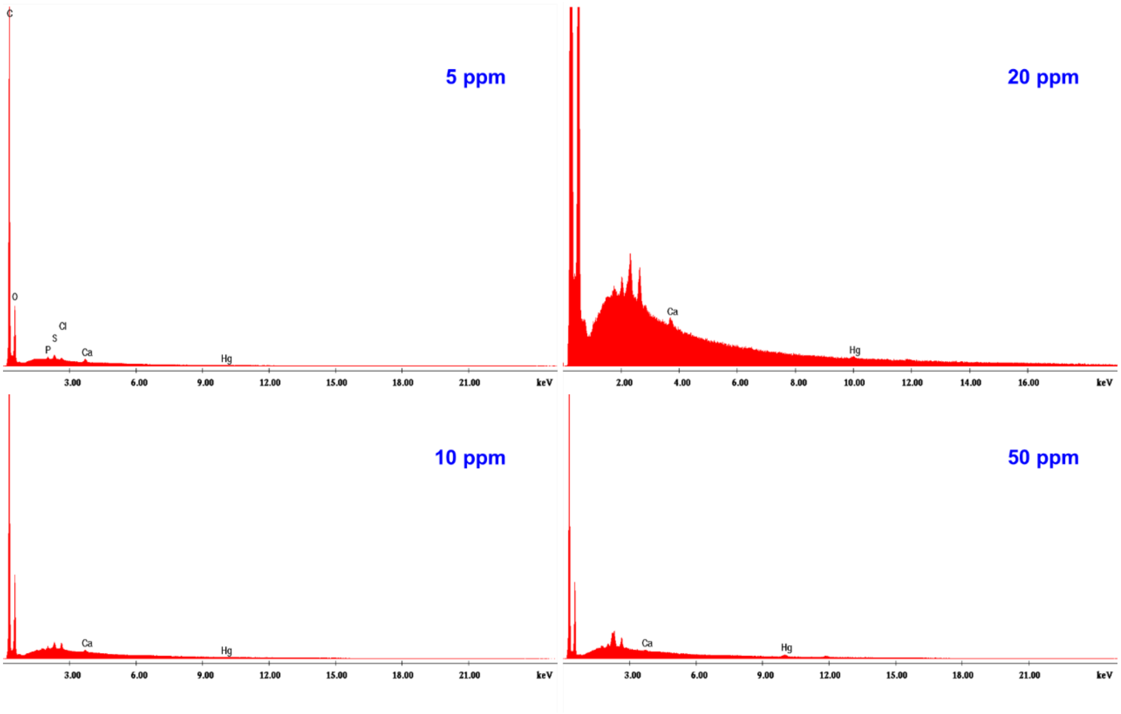

Figure S3. EDS spectra of X-alginate beads after adsorption from solutions with initial Hg^{2+} concentrations equal to 5, 10, 20 and 50 mg·L^{-1}, as indicated.

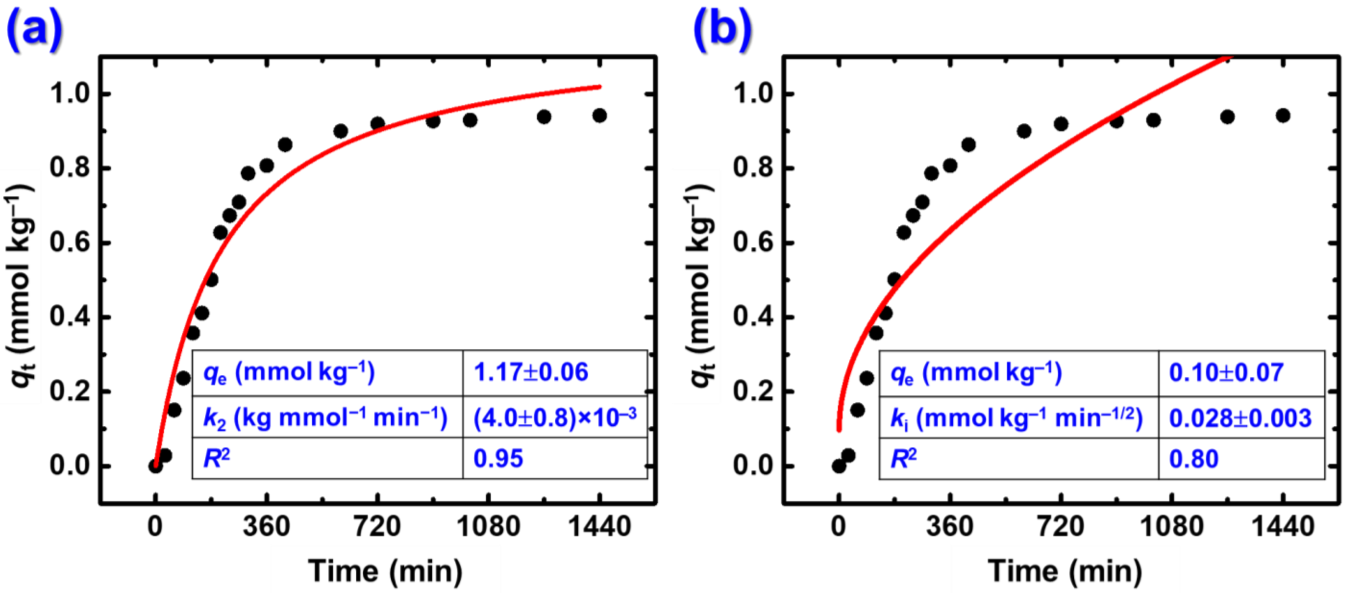

Figure S4. Pseudo-second order (a) and intraparticle diffusion (b) kinetic model fit for Hg^{2+} adsorption on X-alginate aerogel beads.

Table S1. Selected material properties of X-alginate aerogel beads.

Sample [a]	Bulk density ρ_b (g cm^{-3})	Skeletal density ρ_s (g cm^{-3})	Porosity [b] Π (% v/v)	BET surf. area σ (m^2 g^{-1}) [micropore surf. area] [c]	$V_{1.7\text{-}300nm}$ [d] (V_{Total}) [e] (cm^3 g^{-1})	Av. pore diam. [f] ($4V_{Total}/\sigma$) (nm)
**X-alginate**	0.109±0.008	1.65±0.07	93	322 [16]	0.5 (8.6)	7.1 (106)

[a] The concentration of the sodium alginate solution was 2% w/w. [b] Porosity calculated according to the formula: $(\rho_s - \rho_b)/\rho_s$, where ρ_s: skeletal density and ρ_b: bulk density. [c] Micropore surface area *via t*-plot analysis, according to the Harkins and Jura model. [d] Cumulative volume of pores between 1.7 and 300 nm from N_2-sorption data and the BJH desorption method. [e] Total pore volume calculated according to formula: $1/\rho_b - 1/\rho_s$. [f] Calculated by the 4 V/σ method; V was set equal to the maximum volume of N_2 adsorbed along the isotherm as $P/P_o \rightarrow 1.0$. For the number in parentheses, V was set equal to V_{Total} from the previous column.

Table S2. Metal concentrations in the industrial wastewater used in this study.

Metals in wastewater	Concentration (μg L^{-1})
Ni	310
Hg	60.9
Fe	16.8
Cr	13.8
Co	9.82
Sr	6.28
Se	4.31
Al	3.92
Cu	2.04
As	1.92
Zn	1.71
Ba	0.690
V	0.352
Pb	0.282
Rb	0.229
Mn	<0.11
Cd	<0.022
Cs	<0.022
Ti	<0.022
